# Real‐Time MRI of the Chest Wall, Diaphragm, and Lungs in Children

**DOI:** 10.1002/ppul.71133

**Published:** 2025-06-05

**Authors:** Franz Wolfgang Hirsch, Ina Sorge, Christian Roth, Rebecca Anders, Jens Frahm, Dirk Voit, Freerk Prenzel, Martin Lacher, Daniel Gräfe

**Affiliations:** ^1^ Department of Pediatric Radiology University Hospital Leipzig Leipzig Germany; ^2^ Biomedical NMR, Max Planck Institute for Multidisciplinary Sciences Göttingen Germany; ^3^ Department of Pediatrics University Hospital Leipzig Leipzig Germany; ^4^ Department of Pediatric Surgery University Hospital Leipzig Leipzig Germany

**Keywords:** magnetic resonance imaging, radiology, real‐time imaging, sedation, thoracic imaging

## Abstract

**Objective:**

To evaluate the clinical applicability and diagnostic potential of real‐time magnetic resonance imaging (rtMRI) as a sedation‐free, radiation‐free imaging modality for assessing the chest wall, diaphragm, and lungs in children.

**Methods:**

This video‐based narrative review summarizes over four years of clinical experience with rtMRI in pediatric thoracic imaging. Real‐time MRI achieves very high frame rates (up to 50 images per second), effectively minimizing motion artifacts. Representative clinical scenarios—including pneumonia, chest wall tumors, diaphragmatic dysfunction, airway anomalies, and congenital lung malformations—are presented to illustrate the diagnostic capabilities and limitations of rtMRI.

**Results:**

Real‐time MRI enables reliable imaging of thoracic structures in awake and uncooperative children, minimizing motion artifacts and thus eliminating the need for sedation. It provides diagnostic information on conditions such as pneumonia, abscesses, effusions, and neoplastic lesions, using two basic MR contrasts. Diaphragmatic motion and large airway morphology are clearly visualized. Limitations include reduced sensitivity for detecting interstitial lung disease and small pulmonary metastases. Complete thoracic examinations can be performed within 4 minutes, supporting rapid triage and potentially obviating the need for CT or sedated MRI in selected cases.

**Conclusion:**

Real‐time MRI is a promising, child‐friendly alternative for thoracic imaging in pediatrics, particularly for evaluating chest wall abnormalities, diaphragmatic disorders, and pneumonia‐related complications. Broader adoption and vendor‐independent implementation may help establish rtMRI as a routine modality in pediatric chest imaging.

## 1 Introduction

After more than 4 years of clinical application of real‐time MRI (rtMRI) in pediatric thoracic imaging, we aim to provide a comprehensive review of our experiences through a video‐based overview [1].

Real‐time MRI represents a groundbreaking advancement in MR imaging. With frame rates reaching up to 50 images per second, motion artifacts caused by breathing or cardiac activity are markedly decreased. This capability is particularly advantageous for imaging thoracic pathologies, including diaphragmatic disorders, chest wall abnormalities, airway conditions, and most notably, the lungs. The examination is performed during free breathing and without the need for sedation, as even minor macro‐movements by the child do not produce significant artifacts. This makes rtMRI particularly suitable for uncooperative patients, such as young children under 6 years of age [2].

Despite its promising potential, knowledge regarding the applications of rtMRI remains limited. This has motivated us to share our practical experiences and demonstrate the utility of this technique in thoracic imaging (Video 1).

**Video 1:** Thoracic‐lung examination of a 4‐year‐old child with pneumonia in the right lower lobe, visualized in a transverse orientation in a proton density‐weighted volume coverage sequence. Despite breathing and macro‐movements by the child, all anatomical structures are sharply defined. The pneumonic infiltration is distinctly delineated from the surrounding normally aerated lung tissue (http://pedz.de/supplementals/rtmri-lung/Video1.mp4).

The advantages of ultra‐fast MRI for imaging the lungs and thorax are accompanied by certain limitations. While the spatial resolution of 1 × 1 × 3 mm is only slightly reduced compared to standard MRI sequences, it remains lower than that of CT. This limitation is insignificant for visualizing larger structures but reduces the reliability of detecting smaller lesions. Additionally, as with all conventional MR sequences, hyperinflation and interstitial lung diseases (referred to as MR‐minus pathologies) cannot be adequately assessed [3]. Alternative lung‐specific sequences with ultra‐short echo times (TE) are more suitable for these findings, as their extremely short echo time allows them to distinguish even subtle differences between ventilated lung parenchyma and pure air [4, 5, 6].

Despite these constraints, rtMRI has demonstrated high effectiveness in diagnosing conditions characterized by excess proton signals (so‐called MR‐plus pathologies), such as inflammatory or neoplastic diseases, as well as diaphragmatic pathologies. Additionally, non‐contrast‐enhanced vascular imaging of major thoracic vessels in real‐time is emerging as a valuable diagnostic option.

Because rtMRI examinations in infants and young children can typically be performed without sedation, it is frequently employed in our department as a first‐line diagnostic method in early childhood [7].

### 1.1 Why Use Ultra‐Fast Sequences With Reduced Resolution in Pediatric Imaging?

The new AFARA concept in pediatric MRI diagnostics represents a paradigm shift in pediatric radiology. AFARA, or *As Fast As Reasonably Achievable*, departs from the traditional pursuit of achieving the highest possible MRI image quality for every clinical question. Instead, it permits reduced image quality when appropriate, provided it accelerates the examination and still offers sufficient diagnostic confidence to address the clinical query [8, 9]. However, the optimal image quality required for specific diagnostic questions has not yet been universally defined.

Studies indicate that the AFARA approach can substantially reduce the need for sedation in children, particularly for cranial and spinal MRI [7, 9]. The rtMRI of the lung aligns closely with the AFARA concept in many aspects.

The rtMRI technique described here is highly child‐friendly, as it avoids the use of sedation. Additionally, it offers economic advantages, including increased patient throughput and a significant reduction in the use of anesthesia resources for young children [10]. All applications described in this review for thoracic imaging are part of a registry study approved by the local ethics committee (228/20‐ek) and informed consent was obtained from the legal guardians of the patients.

This video‐based review aims to equip readers with the tools to independently evaluate when ultra‐fast rtMRI can already serve as a valid diagnostic tool in the thoracic region. Additionally, the included case studies highlight scenarios where rtMRI may not suffice, and standard MRI or CT imaging remains necessary.

### 1.2 Why Is MRI Now So Fast?

With frame rates of 50 images per second (or up to 100 images per second at lower resolution), real‐time magnetic resonance imaging surpasses even the image frame rates of ultrasound diagnostics [11]. But how is it possible to achieve such high imaging speeds with a conventional magnetic resonance imaging scanner, traditionally known for slow image sequences?

The real‐time magnetic resonance imaging described here is synonymous with the so‐called FLASH 2 technique, which is based on the fast low‐angle shot (FLASH) sequences developed in the 1980s [12, 13]. FLASH sequences dramatically accelerated imaging, making it up to 100 times faster than spin‐echo sequences. In 2010, the same team that developed FLASH introduced FLASH 2 technology, which relies on two key innovations:

#### 1.2.1 Undersampling

Significantly fewer raw data points are acquired than would typically be required for conventional image reconstruction. This enables faster acquisition speeds. However, traditional reconstruction methods, such as Fourier transformations, cannot generate diagnostic‐quality images from such limited data.

#### 1.2.2 Nonlinear Inverse Reconstruction

A novel algorithm reconstructs diagnostic images from heavily undersampled raw data. This algorithm estimates missing raw data points using information from prior images (both spatially and temporally) through advanced mathematical calculations [11].

The combination of these techniques allows MRI to achieve high frame rates with excellent spatial resolution. A selected organ layer can be continuously observed without motion artifacts. In thoracic imaging, for instance, the beating heart can be visualized with high temporal resolution in a single plane, even in cases of arrhythmia, rapid heartbeats, or slight macro‐movements by the child during the examination [14].

For lung imaging, sequential single‐layer imaging would result in gaps due to constant respiratory motion. To address this, FLASH 2 introduced the Volume Coverage (VC) technique. This approach incrementally shifts the slice plane by fractions of a millimeter after each ultra‐fast image acquisition, enabling a complete volume scan within seconds [15]. The resulting stack of slices resembles a CT's thick‐slice multiplanar reconstruction (MPR). For example, with a slice thickness of 3 mm, the plane shifts by only 0.3 mm per acquisition, giving the impression of a 3D image series with an apparent slice thickness of 0.3 mm when viewed. This seamless imaging approach accommodates irregular breathing, providing uninterrupted visualization of structures such as pulmonary blood vessels, even with free and irregular respiration.

An additional advantage of this technique is the reduced Specific Absorption Rate (SAR). Energy deposition in rtMRI is significantly lower than in conventional MRI sequences, consistently maintaining SAR limits safely.

In principle, the FLASH2 real‐time MRI technique is compatible with MRI systems from all manufacturers, as the underlying MRI sequence—a gradient echo sequence—is part of every vendor's portfolio. However, due to significant differences in the implementation of automated processing pipelines across manufacturers, FLASH2 has so far only been realized on Siemens MRI systems (1.5 Tesla and 3 Tesla). There are currently 13 local real‐time MRI installations utilizing the FLASH 2.0 technique, including 10 in Europe and 2 in the United States. As certification for routine clinical use by medical device regulatory authorities (CE and FDA) is still pending, all real‐time MRI examinations of the pediatric thorax presented in this study are part of a registry study approved by the local ethics committee (228/20‐ek).

### 1.3 Advantages and Limitations of Real‐Time MRI in Thoracic Imaging

Traditionally, thoracic compartments in children are examined using respiratory‐triggered and ECG‐triggered MRI sequences [16]. Although MRI protocols vary across institutions, most rely on respiratory triggering. Data acquisition occurs during a quiet expiratory phase, requiring extended examination times and remaining highly sensitive to irregular breathing and motion artifacts. For children under 6 years of age, these protocols often necessitate sedation administered by an anesthesiologist. Even older children may face challenges maintaining calm, steady breathing during triggering, especially in cases of severe lung disease, coughing, dyspnea, or anxiety about the examination [17].

In such situations, rtMRI offers a promising alternative. During the examination, children are gently held by their parents or another accompanying person. The chest coil is supported by a lightweight plastic bridge placed over the thorax, allowing even small children to breathe freely without being encumbered by the coil's weight.

To successfully integrate rtMRI into the diagnostic repertoire of pediatric thoracic imaging, it is essential to identify the clinical questions that can already be addressed with these rapid imaging techniques while also acknowledging their limitations.

### 1.4 Contrasts in Real Time MRI Sequences for Thoracic Applications

While rtMRI images are free from motion artifacts, they present an unfamiliar contrast profile. Due to the mixed weighting of gradient echo sequences, the signal intensity of pathological findings is lower compared to T2‐weighted spin‐echo sequences. Additionally, transient signal loss may occur in certain situations, particularly during rapid breathing, especially with T2/T1‐weighted imaging [10].

The VC technique from the FLASH 2 repertoire is the preferred method for thoracic and lung examinations. Like other FLASH 2 techniques, VC sequences allow for two distinct contrast options: (a) Proton Density (PD) Weighting and (b) T2/T1 mixed weighting [15] (Video 2).

**Video 2:** Visualization of different contrasts in real‐time MRI using a case of Ewing's sarcoma in the right seventh rib (green arrow) with extensive pleural metastases on the mediastinal side of the pleura. The left panel displays Proton Density weighting, while the right panel shows T2 weighting. Notably, the tissue contrast is significantly higher in the T2‐weighted sequence (red arrow) (http://pedz.de/supplementals/rtmri-lung/Video2.mp4).

In our experience, T2/T1 weighting has demonstrated greater diagnostic utility for chest wall and mediastinal conditions due to its superior soft‐tissue contrast, aligning with the preference for T2 weighting in conventional thoracic MRI. Conversely, PD weighting is better suited for lung imaging in a dynamic environment because of its enhanced signal stability and reduced smearing artifacts.

The signal inconsistency observed with T2/T1 weighting during lung imaging stems from the technical characteristics of FLASH 2 sequences, which are steady‐state sequences. In these sequences, the MR signal builds up over multiple excitations and is then sustained [18]. Rapid motion of a structure through the imaging plane disrupts the steady state, leading to signal inconsistencies, particularly in transverse slice orientations. PD weighting, with its lower flip angle, stabilizes the signal more quickly, minimizing signal loss during rapid movement.

A distinct advantage of gradient echo sequences, including rtMRI, is their consistent hyperintense depiction of flowing blood, the so called bright blood imaging [19]. This characteristic can be diagnostically useful, such as in identifying anomalies in aortic arch branches without requiring contrast agents. Additionally, rtMRI has potential for detecting pulmonary embolisms, although the sensitivity and specificity for this application have yet to be fully validated (Video 3).

**Video 3:** Contrast‐free vascular imaging demonstrating a right aortic arch with lusory artery (red arrow) causing mild tracheal compression. Sequential slice shifting is employed, with each position scanned for 3 s. The slightly increased flip angle of 12° enhances the high inflow signal within the blood vessels (http://pedz.de/supplementals/rtmri-lung/Video3.mp4).

On the other hand, the pronounced hyperintense signal of blood vessels can occasionally obscure low‐signal pulmonary findings, such as small bronchopneumonia lesions or minute metastases. Adjusting the flip angle during proton excitation can mitigate the blood's high inflow signal to some extent, but it cannot completely eliminate it [20].

### 1.5 Chest Wall Pathology

Chest wall pathologies in children encompass a range of conditions, including benign abnormalities like pectus excavatum and lymphangioma, as well as malignant tumors such as Ewing's sarcoma, which often originates from the ribs (Video 4).

**Video 4:** Volume coverage sequence in T2/T1‐weighting in a 3‐year‐old girl with Ewing's sarcoma affecting the right fifth rib. The large tumor is clearly visible, along with an expanded intercostal space and rib destruction (indicated by the red arrow) (http://pedz.de/supplementals/rtmri-lung/Video4.mp4).

Both masses and pleural metastases on the parietal pleura are effectively detected using T2/T1 VC weighting. This sequence also provides clear visualization of associated pleural effusions. Unlike lung tissue, the chest wall exhibits significantly reduced movement, which minimizes the described signal loss caused by the absence of steady‐state formation. Given the short sequence duration (10–30 s, depending on thoracic size), chest wall processes should be scanned at least in two orientations (Video 5).

**Video 5:** Real‐time MRI of a 14‐year‐old boy with pectus excavatum using a vacuum suction device. Upper image: Relaxed state without the suction device. Lower image: Funnel elevation following suction application (http://pedz.de/supplementals/rtmri-lung/Video5.mp4).

### 1.6 Diaphragmatic Disorders

Real‐time MRI is ideally suited for diagnosing congenital malformations and other diaphragmatic disorders. VC sequences allow coronal and sagittal imaging of the entire diaphragm within seconds, eliminating the need for sedation. In cases of suspected unilateral diaphragmatic paralysis or to differentiate it from diaphragmatic relaxation, a coronal single‐slice measurement with high temporal resolution is particularly effective. This approach reliably evaluates diaphragmatic amplitude and synchrony, providing more detailed diagnostic information than fluoroscopy or ultrasound (Video 6).

**Video 6:** Real‐time MRI volume coverage sequence in T2/T1 weighting of a postoperative child with right‐sided diaphragmatic paralysis. The right diaphragm is elevated and shows reduced, paradoxical movement compared to the left (http://pedz.de/supplementals/rtmri-lung/Video6.mp4).

### 1.7 Visualization of the Central Airways

The visualization of air within the trachea and main bronchi is reliably achieved using PD‐weighted rtMRI sequences. Signal‐free air is clearly outlined against the signal‐rich mediastinal tissues, enabling assessment of large airways down to the proximal lobar bronchi.

Anomalies and malformations of the trachea and main bronchi can be diagnosed quickly and reliably with rtMRI. Although ultra‐short TE sequences are currently the gold standard for visualizing the trachea and bronchi, they require longer acquisition times (3–8 min), during which the child must remain motionless but can breathe freely [21]. In comparison, rtMRI provides slightly less sharp visualization of the main bronchi in PD weighting, with broader outlines (Video 7).

**Video 7:** A young patient with severe tracheal stenosis. Left: Coronal Ultra Short TE sequence. Right: Real‐time MRI in proton density weighting. The right upper lobe bronchus arising directly from the trachea (a bronchus suis) is highlighted as a variant (green arrow), while a significant tracheal stenosis is visible just above the carina (red arrow) (http://pedz.de/supplementals/rtmri-lung/Video7.mp4).

Another application is the visualization of the tracheal cross‐section during free breathing to assess and quantify tracheomalacia. Free mediastinal air, indicative of tracheal or esophageal perforation, can also be detected with the same reliability as computed tomography. Esophageal perforations may sometimes occur following the ingestion of button batteries, often with a delayed onset (Video 8).

**Video 8:** A young child with esophageal perforation following button battery ingestion. The discharge current often leads to necrosis, resulting in acute or delayed perforations. The esophageal position is clearly defined by the signal‐free nasogastric tube. Free mediastinal air adjacent to the esophagus is readily visible in the proton density‐weighted real‐time MRI (red arrow) (http://pedz.de/supplementals/rtmri-lung/Video8.mp4).

### 1.8 Lung Diseases

Normal lung parenchyma appears signal‐free in rtMRI, both in PD weighting and T2/T1 weighting, similar to conventional T2 spin‐echo sequences [22]. Only high‐signal blood vessels are visible due to the inflow effect, traceable up to the fourth branching order. Pathologies with increased proton density also exhibit increased signal in T2/T1 weighting with rtMRI, although the signal‐to‐noise ratio is lower compared to conventional spin‐echo sequences.

The visibility of pathological findings improves significantly with greater proton density and lesion size. Atelectasis, pneumonia, and pleural effusions are well‐depicted as MR‐plus pathologies in rtMRI. Within pneumonic infiltrates, abscesses can be clearly distinguished based on signal intensity differences. This makes rtMRI particularly valuable for rapid, radiation‐free, and sedation‐free diagnosis that supports therapy decisions in cases of complicated pneumonia (Videos 9 and 10).

**Video 9:** Real‐time MRI (rtMRI) of a 1‐year‐old child with complicated pneumonia. A pleural effusion is visible on the left lateral side (red arrow), while large consolidations appear in the left lower lobe and the right upper lobe (green arrows). Within the right upper lobe infiltrate, a hyperintense abscess is detected (yellow arrow). The hands of the accompanying adult holding the child are visible at the sides—typical for an rtMRI examination (http://pedz.de/supplementals/rtmri-lung/Video9.mp4).

**Video 10:** A young immunodeficient patient with cryptococcal pneumonia. While the diagnosis cannot be sufficiently established from the chest X‐ray (left side), the computed tomography scan reveals a characteristic reverse halo sign. This reverse halo sign is similarly depicted in the proton density‐weighted real‐time MRI (right side) (http://pedz.de/supplementals/rtmri-lung/Video10.mp4).

Interstitial lung diseases such as fibrosis or interstitial inflammation, as well as other proton‐deficient pathologies like cysts or bullae, cannot be reliably diagnosed with rtMRI. They are classified as MR‐minus pathologies, for which new ultra‐short TE (UTE) sequences provide an effective alternative [23, 24, 25].

The role of rtMRI in detecting pulmonary metastases remains inconclusive. While rtMRI offers complete gapless lung coverage, we observed insufficient detection of small metastases measuring 3 mm, and occasionally even 8 mm lesions were missed, though metastases larger than 10 mm were reliably identified. Coronal slice orientation and PD weighting proved more stable for metastasis imaging, but in its current state, rtMRI is not yet suitable for routine metastasis screening (Videos 11 and 12).

**Video 11:** MRI of a young patient with multiple pulmonary metastases from a soft‐tissue sarcoma. Left: Standard MRI in T2 weighting with a scan duration of 4:40 min. Right: Proton density‐weighted real‐time MRI volume coverage sequence. In this case, even small metastases measuring 3 mm in diameter were visible in the real‐time MRI sequence (http://pedz.de/supplementals/rtmri-lung/Video11.mp4).

**Video 12:** MRI of a 2‐year‐old child with pulmonary metastases from a neuroblastoma. The video sequentially presents respiratory‐triggered, T2‐weighted standard MRI sequences, followed by real‐time MRI volume coverage sequences in T2/T1 weighting, and finally the proton density‐weighted sequence. Not all metastases visible in the conventional MRI were detected in the real‐time MRI sequences (http://pedz.de/supplementals/rtmri-lung/Video12.mp4).

For congenital lung abnormalities, the suitability of rtMRI varies depending on the specific entity. For example, lung sequestration, classified as an MR‐plus pathology, can be easily distinguished from signal‐free normal lung tissue due to the signal‐rich, non‐ventilated sequestration tissue. Additionally, contrast‐free angiography of rtMRI can identify the atypical vascular supply and venous drainage, confirming the diagnosis of sequestration. However, the sensitivity and specificity for such diagnoses have not yet been systematically evaluated (Video 13).

**Video 13:** A 7‐day‐old neonate with an intralobar lung sequestration. Left: Ultra‐short TE sequence. Right: Transverse real‐time MRI slices reveal the sequestration artery originating from the aorta (red arrow). Venous drainage into the pulmonary veins is more clearly visualized on subsequent coronal slices (green arrow) (http://pedz.de/supplementals/rtmri-lung/Video13.mp4).

Diagnosing MR‐minus pathologies, such as congenital pulmonary airway malformations (CPAM), is more challenging with MRI [26]. In the early postnatal period, when CPAM cysts are still partially fluid‐filled, rtMRI can sometimes distinguish them from other causes of radiographic opacities. For unclear findings during rtMRI, changing the patient's position from supine to prone can help reveal a redistribution of fluid from dorsal to ventral regions (Videos 14 and 15).

**Video 14:** Neonate with congenital pulmonary airway malformation (CPAM). Starting with a day‐of‐birth radiograph showing nonspecific findings of opacity. First part of the video shows the real‐time volume coverage sequence in T2/T1 weighting taken 2 days later. Partially fluid‐filled lung cavities with air‐fluid levels, characteristic of CPAM type 1 immediately after birth, are visible. Second part of the video shows repositioning the infant to the prone position reveals a redistribution of fluid. The final follow‐up radiograph 5 days later shows the typical air‐filled CPAM pattern after fluid resorption, which is no longer reliably diagnosable with real‐time MRI (http://pedz.de/supplementals/rtmri-lung/Video14.mp4).

**Video 15:** Real‐time MRI volume coverage sequences in a 16‐year‐old patient with cystic fibrosis. Proton density weighting (left) and T2/T1 weighting (right) clearly depict extensive bronchiectasis in the right upper lobe (circles). A narrow atelectasis in the upper lobe is indicated by blue arrows (http://pedz.de/supplementals/rtmri-lung/Video15.mp4).

### 1.9 Summary

Real‐time MRI of the thorax, in its current state of development, provides a child‐friendly approach to assessing thoracic diseases without significant motion artifacts. It allows reliable evaluation of chest wall processes and diaphragmatic disorders. Its use in the mediastinum and lungs should be carefully considered based on specific clinical indications. Real‐time MRI is particularly valuable for diagnosing complications of pneumonia, such as abscesses, and for visualizing atelectasis. However, it is not yet an adequate replacement for respiratory‐triggered lung MRI sequences in cases such as small metastasis detection, interstitial lung diseases, or cysts.

A complete rtMRI examination of the thorax requires only a 4‐min timeslot, making it a viable alternative to chest X‐rays, ultrasound, or even computed tomography in selected cases, provided radiology departments can offer low‐threshold, short‐term access to MRI. In routine practice, rtMRI can be effectively combined with subsequent standard sequences. Children often settle down after initial rtMRI, enabling one to two additional respiratory‐triggered sequences. Adding an ultra‐short TE sequence can complement the imaging by detecting MR‐minus pathologies, such as fibrosis and cysts.

Once rtMRI becomes widely available across manufacturers, it is likely to become a standard option for thoracic imaging in pediatric radiology.

## Author Contributions

**Franz Wolfgang Hirsch:** conceptualization, investigation, funding acquisition, writing – original draft, methodology, validation, writing – review and editing, resources. **Ina Sorge:** writing – review and editing. **Christian Roth:** writing – review and editing. **Rebecca Anders:** writing – review and editing. **Jens Frahm:** writing – review and editing. **Dirk Voit:** writing – review and editing. **Freerk Prenzel:** writing – review and editing. **Martin Lacher:** writing – review and editing. **Daniel Gräfe:** writing – review and editing, investigation, funding acquisition, writing – original draft, supervision, visualization, project administration, data curation.

## Conflicts of Interest

Jens Frahm and Dirk Voit are co‐inventors of a patent and software describing the real‐time MRI technique used here. The other authors declare no conflicts of interest.

## Data Availability

The data that support the findings of this study are available from the corresponding author upon reasonable request.
